# Bionanotechnology and bionanomaterials: John Honek explains the good things that can come in very small packages

**DOI:** 10.1186/1471-2091-14-29

**Published:** 2013-11-04

**Authors:** John F Honek

**Affiliations:** 1 Chemistry, University of Waterloo, 200 University Avenue West, Waterloo, Ontario N2L 3G1, Canada

## Introduction

In addition to acting as Professor and Chair of the Department of Chemistry at the University of Waterloo in Ontario, Canada, and being a member of the Waterloo Institute for Nanotechnology (WIN), John Honek is the Section Editor of the Protein and Enzyme Biochemistry section of *BMC Biochemistry*. Professor Honek’s group has recently moved into looking at the application of multisubunit protein systems and viruses for bionanomaterial fabrication. We therefore saw this as an opportune time to talk to him about the exciting field of bionanomaterials and bionanotechnology, in order to gain a better understanding of just what it involves, and what we can expect from research in this area.

### Questions

### 1. Firstly, what are bionanomaterials and why are they important?

A general definition of bionanomaterials would be molecular materials composed partially or completely of biological molecules (such as antibodies, proteins/enzymes, DNA, RNA, lipids, oligosaccharides, viruses, and cells for example) and resulting in molecular structures having a nanoscale-dimension(s). The resulting bionanomaterials may have potential applications as novel fibers, sensors, adhesives, energy generating and/or harnessing materials, to mention just a few aspects. These types of systems can allow for fabrication of complex devices by self-assembly under mild experimental conditions and in an eco-friendly manner such as at room temperature and in aqueous conditions.

### 2. It can be difficult to imagine the nanoscale on which these technologies operate. Is there a useful analogy that would help readers understand this scale more easily?

The nanoscale is generally meant to encompass the dimensions between 1–100 nanometers. A sheet of regular paper is approximately 100,000 nanometers thick, and the diameter of a gold atom is roughly one-third of a nanometer.

### 3. What sorts of technologies and techniques are being used in bionanomaterial research?

Numerous research fields are intersecting in this area. Disciplines such as biochemistry, materials science, chemistry, immunology, physics, mathematics and engineering are playing major roles in this research. For example, biochemists are realizing that their ability to isolate, characterize and engineer biomolecules such as proteins and antibodies, through recombinant DNA techniques, are essential components to bionanomaterial research. Advanced physical techniques such as electron and helium ion microscopic techniques and nanofabrication protocols are also permitting nanosized device manufacture and analysis.

### 4. We often hear reference to terms such as 'biosensor’ and 'DNA origami’ in relation to bionanotechnology. Can you explain what each of these mean?

A biosensor is a device that is used to detect the presence of an analyte. The device would be comprised of a biological component, frequently the sensing element itself, such as an antibody, and an interface capable of measuring the interaction of the analyte with this sensing element and subsequently generating some type of output signal such as an electrical current.

The term DNA origami is used to describe DNA (deoxyribonucleic acid) folding to construct two- and three-dimensional shapes at the nano-dimensional scale. An important factor involved in this approach is that of complimentary DNA base pairing so that the design of these nanoscale systems must pay attention to this interaction. Recent work on the application of DNA origami to bionanomaterials has been as beacons for detection of target molecules in bionanosensors, such as for the detection of single-nucleotide polymorphisms, and also as molecular building components to prepare DNA-based nanopores for controlling DNA translocation, important in DNA sequence determination.

### 6. Are there any ethical issues or concerns with the development and use of bionanomaterials?

Each individual bionanomaterial, and any material or compound for that matter, needs to be looked at as an individual case. For example, bionanomaterials can be composed of proteins or DNA, and although these biomolecules are “natural” compounds, they may have toxicities ranging from no toxicity to high toxicity. That is the same for molecules in general. There are chemical compounds in nature that are harmless such as glucose, and then there are those molecules like strychnine that are highly toxic. One also needs to consider whether the nanodevice is stable over its use or does it start to decompose. If it is placed in the body, one needs to determine what the decomposition products are and whether they are toxic or not. Biomolecules can be metabolized by the body, so proteins that might be released by the decomposition of a bionanomaterial might be readily metabolized by the body, and no toxicity might result. Particle size of any material can be important if inhalation may occur, but nanoscaled measuring devices or electronic materials can be perfectly safe during their use. However, one always needs to keep the entire life cycle of a material, biomaterial, or bionanomaterial in mind when addressing potential health issues.

### 7. We’ve heard a lot of promising things about graphene since its discoverers were awarded the Nobel Prize in 2010. What makes this material so special?

Graphene is one of the strongest materials that has been studied, yet it is also very light with a square meter layer of this material only having a weight of 0.77 milligrams. It has very high electrical conductivity and high optical transparency; properties allowing for its use in advanced electronic devices such as sensors, as a component of liquid crystal displays and organic materials-based photovoltaics, amongst other advanced materials.

### 8. Does investigating biophysical and biochemical effects at the nanoscale pose particular problems relative to investigations at larger scales?

Frequently the interface interaction between biologicals and inorganic materials can pose a challenge to construct and to study. One of these challenges is to make sure that proper orientation of the biological molecule (such as an antibody) on the surface of, for instance, a carbon nanotube to produce a field-effect transistor, is controlled at the nano-dimensional level. Depending on the system under investigation, single molecules of proteins for example are important, whereas for larger scales, the aggregate properties of many biological molecules are the focus of investigation. Applications of redox proteins in the area of bioelectronics, is another area where single biomolecules need to be properly positioned and interfaced to several non-biological components of a device.

### 9. What does the future hold for bionanomaterials in biochemistry and biophysics?

The exquisite control that biochemists have over biomolecular structure, with the ability to position a particular residue/functional group in a predetermined position in three-dimensional space, is already yielding important contributions, such as the protein engineering of the outer surfaces of nano-dimensional virus structures to display antigens that are useful in vaccine development. As well, the ability to utilize nanofabrication methods to provide exactly positioned sensing elements, such as modified nanopores, has allowed for the production of multiplexed biosensors capable of multianalyte detection and quantitation in a miniaturized device - an important contribution in the area of pathogen sensing. Furthermore, the attachment of other components such as quantum dots and gold nanoparticles to biomolecules is already having an impact on our knowledge of cellular biophysics, and extending our knowledge of protein-protein interaction networks.

